# Determinants of Smoking Status in a Sample of Outpatients Afferent to a Tertiary Referral Hospital

**DOI:** 10.3390/ijerph16214136

**Published:** 2019-10-27

**Authors:** Alessandro Radaeli, Matteo Nardin, Danila Azzolina, Mario Malerba

**Affiliations:** 1 ASST Spedali Civili di Brescia, Department of Emergency, University, 25123 Brescia, Italy; alessandro.radaeli@gmail.com; 2 ASST Spedali Civili di Brescia, Department of Internal Medicine, 25123 Brescia, Italy; matteo.nardin.89@gmail.com; 3 Department of Translational Medicine, University of Piemonte Orientale, 28100 Novara, Italy; danila.azzolina@uniupo.it

**Keywords:** smoking, COPD, survey

## Abstract

The identification of determinants of attempts to quit smoking and quitting smoking success is crucial for effective smoking prevention and/or cessation programs. Thus, here we have conducted a survey to determine the sociodemographic characteristics of tobacco use and the potential determinants of quitting smoking among a population of 140 subjects—101 smokers and 39 ex-smokers—referred to our clinic for respiratory diseases. Subject characteristics included demographic data, employment and education status, respiratory disease family history, smoking habits, life habits, diet, alcohol intake, and physical activity. In comparison with former smokers, active smokers were younger, lived with at least one smoking family member, and were more frequently exposed to passive smoke. They also displayed a higher coffee consumption, a higher frequency of in-between-meal snacks, and a lower chronic obstructive pulmonary disease (COPD) prevalence. In comparison with subjects who had never attempted to quit smoking, individuals who had attempted to quit smoking were younger, had a lower pack-year median, consumed a higher amount of coffee and alcohol, and conducted regular physical activity. Determinants of successful smoking cessation were older age, lower passive smoking exposure and daily coffee intake, and COPD diagnosis. Overall, our findings underscore the importance of health education in fostering successful smoking cessation in respiratory disease patients.

## 1. Introduction

In recent years, a significant reduction in cigarette smoking prevalence has been observed worldwide. Among U.S. adults, smoking prevalence has decreased from 21% in 2005 to 15% in 2015 [1]. In Italy, smoking prevalence is relatively high compared to that of other developed countries, including the U.S. [1]. In 2013, 20.9% of the Italian population over the age of 14 years was composed of active smokers, and in 2016 the smoking prevalence among adults was estimated to be at 19.8% [2,3].

Cigarette smoking represents one of the main causes of death in the world, being responsible for about half a million deaths annually, and tobacco use is responsible for 30% of all cancer deaths in the USA [4,5]. Chronic obstructive pulmonary disease (COPD), whose main cause is cigarette smoking, represents the fourth highest cause of death in the world, and it is projected to be the third leading cause of death by 2020 [6].

In this scenario, smoking cessation programs should constitute one of the priorities of health policies because they would reduce the incidence of smoke-related diseases, thereby improving peoples’ quality of life, while lowering the burden of national health care costs. However, due to the complexity of cigarette smoking behavior, it appears quite challenging to devise effective new strategies for smoking cessation. Even though a significant proportion of adult cigarette smokers are actively trying to quit smoking, the percentage of success in quitting is quite low [7]. For example, recent studies have shown that about 53% of current smokers tried to quit smoking in 2018, but a year later only 6% of smokers were successful in achieving their goal, with the vast majority of them relapsing within the first 5 to 10 days from the quit attempt [8,9].

The identification of determinants of quit attempts and success of quitting smoking is therefore crucial to effectively target intervention programs aimed at eradicating tobacco use. In this regard, previous studies have shown that, alongside nicotine addiction, social and psychological features play a major role in the complex decision process to quit without relapse [10]. However, some concerns have been raised by other studies investigating predictors of quitting smoking, probably due to the limited number of determinants investigated per single study and reduced comparability between them [11].

Although a number of studies have assessed the prevalence of smoking and the determinants of tobacco smoking in different populations in Italy [12], there is only scarce information on the characteristics associated with “smoking behavior” and “quitting smoking decisions” in subjects with respiratory symptoms attending respiratory disease clinics. Therefore, here we have conducted a survey to identify the characteristics of tobacco use and the potential determinants of quitting smoking in a population of smokers and former smokers referred to our clinic for respiratory diseases in Italy. 

Altogether, our findings identify the factors involved in smoking cessation in subjects with respiratory symptoms, which could help develop public health interventions able to aid these subjects while quitting smoking. 

## 2. Materials and Methods

### 2.1. Study Design and Setting

The survey was conducted from January 2016 to December 2016. A questionnaire on smoking habits was administered to a consecutive population of active smokers or former smokers referred to our Respiratory Diseases Unit at the Department of Internal Medicine, Hospital Spedali Civili and University of Brescia, Italy. Due to different medical reasons, some of the patients had already been admitted to our unit, whereas others had been referred to our unit by specialists or general practitioners.

A total of 180 questionnaires were administered, of which 140 were completed. 

A smoking subject was defined as a person who had smoked at least one cigarette during the past 30 days, whereas an ex-smoker was defined as a person having previously smoked at least 100 cigarettes, but not having smoked during the past 30 days before the survey.

The questionnaire, available in the supplementary material section, included the following data: demographic data—i.e., sex and age—, employment and education status, family history for respiratory diseases, characteristics of the smoking habit—i.e., pack-years, presence of smokers in the family or at work, attempts to quit smoking, and type of pharmacological support received to help quit smoking—, life habits, diet and alcohol intake, and physical activity. In addition, we collected the past medical history of each patient together with their respiratory functional data, other respiratory tests, such as chest radiography or induced sputum (if present), and clinical symptoms. We also recorded the information relative to the prescribed drug therapy. 

Alcohol intake was assessed by the AUDIT-C questionnaire. Low alcohol consumption (LAC) was defined as having an AUDIT-C value < 4 in males and < 3 in females [13].

All study participants voluntarily signed an informed consent form. The study was conducted in accordance with the principles of the Helsinki Declaration and was approved by the Local Ethics Institutional Board.

### 2.2. Statistical Analysis

Descriptive statistics of data according to groups were collected. Data are reported as median (I and III quartiles), mean±standard deviation for continuous variables, and percentages (absolute numbers) for qualitative variables. The Wilcoxon–Kruskal–Wallis test was performed for continuous variables, while the Pearson chi-square test analyzed categorical variables.

Univariable logistic regression models were computed, considering separate predictors of “attempt to quit smoking” and “success of quitting attempts”. A Boruta feature selection algorithm was employed to identify the most important predictors of “attempt to quit smoking” and “success of quitting attempts”. 

The relevant predictors were considered for multivariable logistic regression model estimation. Computations were performed using R 3.3.3 [14] (R Foundation for Statistical Computing, Vienna, Austria) with rms [15] packages.

## 3. Results

We analyzed 140 consecutive patients, of whom 45 were females (32%), and 95 were males (68%), with a mean age of 55 ± 15 years. In this cohort, 101 subjects were active smokers, while 39 were ex-smokers. The main baseline differences are shown in Table 1. The active smoker group had a lower mean age compared to that of ex-smokers (51 ± 15 vs. 65 ± 13 years, respectively; *p* < 0.001), was more frequently exposed to passive smoke (78% vs. 54%; *p* = 0.004), and more often reported at least one relative smoker (67% vs. 38%; *p* = 0.002). Active smokers consumed not only a higher number of coffees per day (i.e., more than three coffees/day) compared to ex-smokers (50% vs. 15%, respectively; *p* < 0.001), but they also had more in-between-meal snacks (33% vs. 15%; *p* = 0.04). Furthermore, active smokers were less likely to be diagnosed with COPD compared to subjects who quit smoking (33% vs. 66%, respectively; *p* < 0.001).

Before the survey, asthma or COPD had already been diagnosed in 22 and 50 patients, respectively. After clinical and spirometric evaluation, 18 patients were newly diagnosed with asthma, whereas 7 patients were diagnosed with COPD. Thus, our sample was composed of 43 patients without asthma and COPD (31.4%), 40 patients with asthma (28.6%), and 57 with COPD (40%).

The prevalence of asthma in smokers was similar to that detected in ex-smokers (28% vs. 29%, respectively), while COPD was more frequently observed in the ex-smoker group (62% vs. 33%; *p* = 0.002).

Next, in the subgroup of active smokers (n = 101), we compared patients who had attempted to quit smoking (AQ) (n = 60; 59.5%) with patients who had never done so (NAQ) (n = 41; 40.5%). AQs were younger than NAQs (mean age of 48 ± 13 vs. 57 ± 15 years, respectively; *p* = 0.003) and were more often asthmatic (38% in AQs vs. 15% in NAQs; *P* = 0.01). In addition, AQs reported a lower pack-year rate than NAQs (22 ± 12 vs. 37 ± 24 pack-years, respectively; *p* < 0.001), had a higher number of coffees per day (>3 coffees/day) (62% in AQs vs. 34% in NAQs; *p* = 0.007), and consumed fewer alcoholic drinks (34% in AQs vs. 15% in NAQs; *p* = 0.01) (Table 2). Having in-between-meal snacks was associated with a lower frequency of attempts to quit smoking (44% in NAQs vs. 25% in AQs; *p* = 0.05). Doing at least one hour of physical activity per week was more frequent in AQs than NAQs (33% vs. 17%, respectively; *p* = 0.07). 

We identified “number of coffees per day” as an independent predictor of “attempts to quit smoking” (OR [95%CI] = 3.1 [1.37–7.26], *p* = 0.007). On the other hand, “eating in-between-meal snacks” (OR [95%CI] = 0.48 [0.18–0.99], *p* = 0.049), and high intake of alcoholic beverages (OR [95%CI] = 0.34 [0.1–1.4], *p* = 0.063) were found to be independent inverse predictors of “attempts to quit smoking” (Table 3).

Moreover, we investigated patients who had attempted to quit smoking at least one time according to success or relapse. The main differences are shown in Table 4.

Mean age was a success factor for quitting smoking—older subjects were significantly more successful in quitting smoking than younger subjects (65 ± 13 vs. 48 ± 13, respectively) (*p* < 001). Lower frequency of passive smoking was associated with success in quitting smoking (17% vs. 41% in successful quitters (SQs) and nonsuccessful quitters (NSQs), respectively; *p* = 0.01). SQs reported less daily consumption of coffees/day (<3 coffees/day) (38% in SQs vs. 84% in NSQs; *p* < 0.001). Surprisingly, the SQ group reported a higher median pack-year rate compared to that of NSQs (33 ± 15 vs. 22 ± 12 in SQs vs. NSQs, respectively; *p* > 0.01), probably due to the fact that SQs, being older than the NSQs, had smoked for a more prolonged time period. On pulmonary function tests, diagnosis of COPD and bronchial obstruction were more frequently found in SQs than NSQs.

Multivariable logistic regression was performed to estimate the success of quitting attempts.

Among smokers who had tried to quit, a greater tendency to relapse was observed in subjects having more coffees per day (Table 4, Table 5). Furthermore, the same tendency was observed in younger subjects and in individuals with higher pack-year consumption during a previous smoking period or in subjects with a passive smoke history.

We identified coffee consumption (>3 coffees/day) as a direct independent predictor of relapse (OR [95%CI] = 8.69 [3.14–28.56], *p* < 0.001). Moreover, age (OR [95%CI] = 0.91 [0.87–0.95], *p* < 0.001) was found to be an independent inverse predictor of relapse—that is, younger subjects were more prone to relapse. 

There was a greater frequency of smokers in families with at least one smoking component (Table 1). Moreover, subjects seemed to be more prone to relapse when reporting other smoking family members (Table 4, Table 5).

A considerable proportion of subjects (35–38%) availed themselves of pharmacological support (Table 1). However, according to our data, this aspect did not appear to affect smoking cessation success—42% of ex-smokers vs. 35% of active smokers used pharmacological support for quitting smoking (Table 1). In our sample, gender and education and employment status did not have any significant effect on the following variables: “active smoker”, “ex-smoker”, “attempts to quit smoking”, “success of quitting smoking”, and “relapse”.

## 4. Discussion

This survey is one of only a few analyzing the characteristics of the smoking habit and the potential determinants of quitting smoking in a group of subjects referred to a clinic for evaluation of respiratory symptoms. Our aim was to determine the smoking habit characteristics of these patients, as well as the determinants of their willingness to quit smoking and the success or failure of such attempts.

Previous studies on determinants of quitting smoking in various populations have reported conflicting findings [12], and some concerns have been raised on the effective ability of surveys to understand the dynamic psychological and physical changes occurring in smokers trying to quit [11]. These discrepancies could be probably ascribed to the fact that habits are linked to cultural factors, and therefore vary greatly among countries.

In particular, previous studies have shown a significant correlation between the efficacy of quitting smoking, self-reported cigarette consumption per day [16,17,18], and the degree of nicotine dependence [19,20,21]. Others have reported a direct relationship between smoking cessation and gender [22,23,24], first cigarette use [22,23,25], years of smoking addiction [26], having a smoking parent [27,28,29], education level [30,31,32], high BMI [33,34,35], previous attempt to quit smoking [23], and employment [18,34]. 

In keeping with the literature, here we show that active smokers are generally younger and more exposed to passive smoking than ex-smokers, suggesting that passive smoking in the home environment and low perception of the importance of a healthy lifestyle in younger individuals are relevant determinants of smoking behavior. Concerning age, our data further supports the notion that older subjects are generally less inclined to relapse once they have quit smoking in comparison with younger subjects. With regard to the aspects of passive smoking and the presence of smokers among family members, our data confirm that the habit of being exposed to passive smoking and closeness to smokers are negatively associated with smoking cessation.

Another important observation emerging from our survey is the negative influence of consumption of in-between-meal snacks, alcohol, and coffee on the smoking habit. In particular, the consumption of more than three cups of coffee is associated with not only an active smoker status but also more attempts to quit smoking, suggesting that drinking coffees is a surrogate of smoking a cigarette. Fittingly, if those individuals who had attempted at least once to quit smoking drank three or more coffees a day, they were more likely to relapse. Furthermore, eating in-between-meal snacks was more frequently found in active smokers who had fewer attempts to quit smoking, Moreover, subjects who had tried to quit eating snacks had a higher relapse frequency. The majority of subjects consuming high levels of alcohol (no LAC) reported fewer attempts to quit smoking, whereas the subjects who attempted to quit showed a tendency (not statistically significant) to relapse (Table 4 and Table 5). Given the nature of the study, it is not possible to formulate a hypothesis on the cause–effect relationship between alcohol, coffee and snack consumption, and the smoking habit. It is however tempting to speculate that these determinants when simultaneously present in a person may reinforce the habit of "smoking addiction". 

Another well-established issue in the literature concerns the diagnosis of COPD, which is associated with the tendency to quit smoking. This is probably related to the fact that the willingness to quit smoking arises primarily from the worsening of a subject’s quality of life due to persistent cough and/or dyspnea, and secondarily from the subject’s awareness of the importance of preserving its state of health after being diagnosed with respiratory disease.

We did not observe any statistical significance regarding the demographic data of gender, education, or employment status, probably due to the rather inhomogeneous and low-number population of the study.

### Study Limitations

The use of the electronic cigarette was not inquired, but according to epidemiological data, in Italy, the use of the electronic cigarette is of little importance and concerns only 1.3% of the general population and 3.7% of smoking subjects [1].

Although comparison with non-smoking subjects referred to our center could have been interesting, we chose to mainly focus on smokers and former smokers.

Other validated instruments have been proposed in the literature [36] in order to evaluate smoking habits among respiratory unit outpatients. However, considering the specific research clinical context, we decided to administer a concise version of the questionnaire. The final version of the administered questionnaire was approved by three independent investigators. 

## 5. Conclusions

Our findings highlight the central role of health education in fostering smoking cessation in subjects with respiratory symptoms. In particular, we show that reduced consumption of alcoholic beverages and coffee and lack of exposure to secondhand smoke are important determinants of quitting smoking in respiratory disease patients. Thus, our findings underscore the importance of promoting conducting a healthy lifestyle among younger people, who quite often perceive as "familiar and normal" their exposure to cigarette smoking. Finally, our data constitute the rationale for the design and development of smoking cessation programs.

## Figures and Tables

**Table 1:** Patients’ characteristics according to smoking status.

Variable	Ex-Smokers	Smokers	All Subjects	*p*-Value
	(N = 39)	(N = 101)	(N = 140)	
Age (years)	58/66/76 65+/−13	39/51/61 51+/−15	41/56/67 55+/−15	<0.001
Gender: M	72% (28)	66% (67)	68% (95)	0.5
Pack-years	20/35/44 34+/−17	15/23/40 28+/−19	15/25/40 30+/−19	0.01
Start age (years)	17/20/22 20+/−5	18/20/23 21+/−5	18/20/23 21+/−5	0.3
Passive smoke exposure	54% (21)	78% (79)	71% (100)	0.004
Smoke exposure at work	36% (14)	36% (36)	36% (50)	1
No. of smoking relatives	0.8/1.0/2.0 1.2+/−1.0	1.0/1.0/2.0 1.2+/−0.7	1.0/1.0/2.0 1.2+/−0.7	0.9
No. of smoking cohabitants	0.0/1.0/1.0 0.8+/−0.8	0.0/1.0/1.0 0.7+/−0.6	0.0/1.0/1.0 0.8+/−0.6	1
No. of smoking coworkers	0/1/3 1+/−2	0/1/3 1+/−2	0/1/3 1+/−2	0.9
Previous quit attempts	82% (32)	59% (60)	66% (92)	0.01
Any smoking relative (%): At least one	38% (15)	67% (68)	59% (83)	0.002
None	62% (24)	33% (33)	41% (57)	
Any smoking cohabitant (%): At least one	31% (12)	49% (49)	44% (61)	0.06
None	69% (27)	51% (52)	56% (79)	
Pharmacological help	42% (14)	35% (21)	38% (35)	0.5
Education:	18% (7)	25% (25)	23% (32)	0.8
high school graduation				
primary school	3% (1)	2% (2)	2% (3)	
university degree	23% (9)	25% (25)	24% (34)	
secondary school	56% (22)	49% (49)	51% (71)	
Eating in-between-meal snacks (%)	15% (6)	33% (33)	28% (39)	0.04
No. of coffees per day:				
less than three	85% (33)	50% (50)	59% (83)	<0.001
more than three	15% (6)	50% (51)	41% (57)	
LAC	15% (6)	29% (29)	25% (35)	0.1
No. of times/week doing physical activity	0.6+/−1.6	0.8+/−1.5	0.7+/−1.5	0.3
Total hours of activity: At least one	18% (7)	27% (27)	24% (34)	0.3
Allergies	21% (8)	19% (19)	20% (27)	0.8
Ischemic heart disease	9% (3)	7% (7)	7% (10)	0.8
Hypertension	44% (16)	37% (37)	39% (53)	0.5
Diabetes mellitus	20% (7)	13% (13)	15% (20)	0.3
Neoplasms	17% (6)	17% (17)	17% (23)	0.9
Diagnosis of COPD	62% (24)	33% (33)	41% (57)	0.002
FEV1 (% predicted)	56/65/75 67+/−16	68/81/92 79+/−17	62/78/90 76+/−18	<0.001
FVC (% predicted)	89/ 98/108 98+/−13	92/102/111 102+/−12	90/101/110 101+/−13	0.2
FEV1/FVC (%)	54/64/75 65+/−16	65/82/90 77+/−14	63/77/88 74+/−16	<0.001
Diagnosis of asthma	28% (11)	29% (29)	29% (40)	1

LAC: low alcoholic consumption; COPD: chronic obstructive pulmonary disease; FEV1: forced expiratory volume at the first second; and FVC: forced vital capacity. Continuous data are reported as median (I, III quartiles); categorical data are reported as percentages and absolute frequencies. Wilcoxon-type and Pearson chi-square tests were performed for continuous and categorical variables, respectively.

**Table 2:** Patients’ characteristics in the active smoker group according to previous attempts to quit smoking.

Variable	No. of Previous Attempts	At Least One Previous Attempt	Combined	*p*-Value
	(N = 41)	(N = 60)	(N = 101)	
Age (years)	47/59/68 57+/−15	39/44/56 48+/−13	39/51/61 51+/−15	0.03
Gender: M	76% (31)	60% (36)	66% (67)	0.1
Pack-years	20/30/47 37+/−24	11/19/30 22+/−12	15/23/40 28+/−19	<0.001
Start age (years)	18/20/22 21+/−4	20/20/25 22+/−5	18/20/23 21+/−5	0.3
Passive smoke exposure	71% (29)	83% (50)	78% (79)	0.1
Smoke exposure at work	39% (16)	33% (20)	36% (36)	0.6
No. of smoking relatives	1.0/1.0/2.0 1.2+/−0.7	1.0/1.0/2.0 1.3+/−0.6	1.0/1.0/2.0 1.2+/−0.7	0.5
No. of smoking cohabitants	0.2/1.0/1.0 0.7+/−0.5	0.0/1.0/1.0 0.7+/−0.6	0.0/1.0/1.0 0.7+/−0.6	0.8
No. of smoking coworkers	0/2/3 2+/−2	0/1/3 1+/−1	0/1/3 1+/−2	0.6
Any smoking relative (%): At least one	59% (24)	73% (44)	67% (68)	0.1
Any smoking cohabitant (%): At least one	46% (19)	50% (30)	49% (49)	0.7
Pharmacological support	0% (0)	36% (21)	35% (21)	0.3
Education:				
high school graduation	20% (8)	28% (17)	25% (25)	0.7
primary school	2% (1)	2% (1)	2% (2)	
university degree	24% (10)	25% (15)	25% (25)	
secondary school	54% (22)	45% (27)	49% (49)	
Eating in-between-meal snacks (%)	44% (18)	25% (15)	33% (33)	0.05
No. of coffees per day:				
More than three	34% (14)	62% (37)	50% (51)	0.07
Alcoholic drink				
No LAC	34% (14)	15% (9)	23% (23)	0.02
No. of times/week doing physical activity	0.0/0.0/0.0 0.6+/−1.4	0.0/0.0/2.0 0.9+/−1.6	0.0/0.0/1.0 0.8+/−1.5	0.1
Total hours of activity: At least one	17% (7)	33% (20)	27% (27)	0.07
Diagnosis of COPD	41% (17)	27% (16)	33% (33)	0.1
Diagnosis of asthma	15% (6)	38% (23)	29% (29)	0.01

LAC: low alcoholic consumption; COPD: chronic obstructive pulmonary disease; FEV1: forced expiratory volume at the first second; and FVC: forced vital capacity. Continuous data are reported as median (I, III quartiles); categorical data are reported as percentages and absolute frequencies. Wilcoxon-type and Pearson chi-square tests were performed for continuous and categorical variables, respectively.

**Table 3:** Monovariable and multivariable logistic regression estimate (OR 95% CI) in active smokers for the previous attempt to quit smoking.

Variable	Monovariable Logistic Regression		Multivariable Logistic Regression	
	OR 95% CI	*p*-Value	OR 95% CI	*p*-Value
Age (years)	0.96 (0.93; 0.99)	0.004	0.97 (0.92; 1.02)	0.226
Gender M	0.48 (0.19; 1.14)	0.106	-	-
Pack-years	0.95 (0.92; 0.98)	0.001	0.97 (0.93; 1)	0.088
Passive smoke exposure: Y	2.07 (0.8; 5.49)	0.136	-	-
Start age (years)	1.04 (0.95; 1.14)	0.396	-	-
Smoke exposure at work: Y	0.78 (0.34; 1.79)	0.558	-	-
No. of smoking colleagues	0.91 (0.66; 1.24)	0.529	-	-
Education: primary school	0.47 (0.02; 12.92)	0.61	-	-
university degree	0.71 (0.22; 2.25)	0.556	-	-
secondary school	0.58 (0.2; 1.56)	0.287	-	-
No. of smoking relatives	1.3 (0.64; 2.81)	0.476	-	-
Any smoking relative (%): None	0.51 (0.22; 1.19)	0.122	-	-
No. of smoking cohabitants	1.03 (0.44; 2.42)	0.953	-	-
Any smoking cohabitant (%): None	0.86 (0.39; 1.91)	0.718	-	-
Snacks (%) Y	0.43 (0.18; 0.99)	0.049	0.18 (0.05; 0.54)	0.004
No LAC	0.34 (0.13; 0.88)	0.028	0.34 (0.1; 1.04)	0.063
No. of times/week doing physical activity	1.17 (0.89; 1.6)	0.286	-	-
Physical activity: None	0.41 (0.15; 1.05)	0.074	-	-
Respiratory diagnosis: Asthma	3.63 (1.39; 10.77)	0.012	-	-
Respiratory diagnosis: COPD	0.51 (0.22; 1.19)	0.122	-	-
FEV1 (% predicted)	1 (0.98; 1.03)	0.771	-	-
FVC (% predicted)	0.99 (0.95; 1.02)	0.483	-	-
FEV1/FVC (%)	1.02 (0.99; 1.05)	0.171	-	-

LAC: low alcoholic consumption; COPD: chronic obstructive pulmonary disease; FEV1: forced expiratory volume at the first second; and FVC: forced vital capacity.

**Table 4:** Characteristics of patients with at least one previous attempt to quit smoking according to success or relapse.

Variable	Successful Quitters	Unsuccessful	Combined	*p*-Value
Age (years)	59/66/75 65+/−13	39/44/56 48+/−13	40/53/66 54+/−15	<0.001
Gender: M	72% (23)	60% (36)	64% (59)	0.3
Pack-year	21/34/43 33+/−15	11/19/30 22+/−12	14/24/36 26+/−14	<0.001
Start age (years)	18/20/21 20+/−5	20/20/25 22+/−5	18/20/24 21+/−5	0.2
Passive smoke exposure	59% (19)	83% (50)	75% (69)	0.01
Smoke exposure on work	38% (12)	33% (20)	35% (32)	0.7
No. of smoking relatives	1.0/1.0/2.0 1.3+/−1.0	1.0/1.0/2.0 1.3+/−0.6	1.0/1.0/2.0 1.3+/−0.7	0.9
No. of smoking cohabitants	0.0/1.0/1.0 0.8+/−0.9	0.0/1.0/1.0 0.7+/−0.6	0.0/1.0/1.0 0.8+/−0.7	0.8
No. of smoking coworkers	0/1/2 1+/−2	0/1/3 1+/−1	0/1/3 1+/−1	0.9
Any smoking relative (%): At least one	44% (14)	73% (44)	63% (58)	0.05
Any smoking cohabitant (%): At least one	34% (11)	50% (30)	45% (41)	0.2
Pharmacological support	45% (14)	36% (21)	39% (35)	0.4
Education:				
high school graduation	16% (5)	28% (17)	24% (22)	0.5
primary school	3% (1)	2% (1)	2% (2)	
university degree	22% (7)	25% (15)	24% (22)	
secondary school	59% (19)	45% (27)	50% (46)	
Eating in-between-meal snacks (%)	16% (5)	25% (15)	22% (20)	0.3
No. of coffees/day:				
More than three	16% (5)	62% (37)	46% (42)	<0.001
Alcoholic drink				
No LAC	9% (3)	15% (9)	13% (12)	0.4
No. of times/week doing physical activity	0.0/0.0/0.0 0.7+/−1.7	0.0/0.0/2.0 0.9+/−1.6	0.0/0.0/1.0 0.8+/−1.6	0.2
Total hours of activity: At least one	19% (6)	33% (20)	28% (26)	0.1
Diagnosis of COPD	62% (20)	27% (16)	39% (36)	<0.001
FEV1 (% predicted)	59/65/75 68+/−17	70/81/92 80+/−16	62/76/90 76+/−17	0.01
FVC (% predicted)	89/ 98/109 98+/−13	89/102/110 101+/−12	89/100/110 100+/−12	0.5
FEV1/FVC (%)	55/64/75 65+/−16	75/82/90 79+/−13	63/77/86 74+/−16	<0.001
Diagnosis of asthma	25% (8)	38% (23)	34% (31)	0.2

LAC: low alcoholic consumption; COPD: chronic obstructive pulmonary disease; FEV1: forced expiratory volume at the first second; and FVC: forced vital capacity. Continuous data are reported as median (I, III quartiles); and categorical data are reported as percentages and absolute frequencies. Wilcoxon-type and Pearson chi-square tests were performed for continuous and categorical variables, respectively.

**Table 5:** Monovariable and multivariable logistic regression estimate (OR 95% CI) in patients with at least one previous attempt to quit smoking according to success or relapse.

Variable	Monovariable Logistic Regression		Multivariable Logistic Regression	
	OR 95% CI	*p*-Value	OR 95% CI	*p*-Value
Age (years)	0.91 (0.87; 0.95)	<0.001	0.93 (0.87; 0.98)	0.009
Gender M	0.59 (0.22; 1.45)	0.26	-	-
Pack-years	0.94 (0.91; 0.97)	0.001	0.99 (0.94; 1.05)	0.745
Passive smoke exposure: Y	3.42 (1.3; 9.33)	0.014	-	-
Start age (years)	1.05 (0.96; 1.16)	0.27	-	-
Smoke exposure at work: Y	0.83 (0.34; 2.07)	0.69	-	-
No. of smoking colleagues	0.98 (0.69; 1.42)	0.916	-	-
Education: primary school	0.29 (0.01; 8.33)	0.415	-	-
university degree	0.63 (0.16; 2.39)	0.5	-	-
secondary school	0.42 (0.12; 1.27)	0.139	-	-
No. of smoking relatives	0.91 (0.44; 1.95)	0.806	-	-
Any smoking relative (%): None	0.28 (0.11; 0.69)	0.006	0.37 (0.1; 1.24)	0.113
No. of smoking cohabitants	0.83 (0.38; 1.83)	0.63	-	-
Any smoking cohabitant (%): None	0.52 (0.21; 1.26)	0.153	-	-
Snacks (%) Y	1.8 (0.62; 6.04)	0.303	-	-
No. of coffees/day: more than three	8.69 (3.14; 28.56)	<0.001	5.4 (1.58; 21.59)	0.01
No LAC	1.71 (0.47; 8.14)	0.449	-	-
No. of times/week doing physical activity	1.08 (0.83; 1.48)	0.58	-	-
Respiratory diagnosis: Asthma	1.86 (0.74; 5.06)	0.201	-	-
Respiratory diagnosis: COPD	0.22 (0.08; 0.54)	0.001	0.84 (0.14; 5.6)	0.855
FEV1 (% predicted)	1.04 (1.01; 1.08)	0.004	0.97 (0.89; 1.04)	0.398
FVC (% predicted)	1.02 (0.98; 1.06)	0.368	-	-
FEV1/FVC (%)	1.06 (1.03; 1.1)	<0.001	1.04 (0.95; 1.14)	0.462

LAC: low alcoholic consumption; COPD: chronic obstructive pulmonary disease; FEV1: forced expiratory volume at the first second; FVC: forced vital capacity; and Tiff: Tiffenau index.
